# Axial and Radial Forces of Cross-Bridges Depend on Lattice Spacing

**DOI:** 10.1371/journal.pcbi.1001018

**Published:** 2010-12-02

**Authors:** C. David Williams, Michael Regnier, Thomas L. Daniel

**Affiliations:** 1 Department of Physiology and Biophysics, University of Washington, Seattle, Washington, United States of America; 2 Department of Bioengineering, University of Washington, Seattle, Washington, United States of America; 3 Department of Biology, University of Washington, Seattle, Washington, United States of America; University of California San Diego, United States of America

## Abstract

Nearly all mechanochemical models of the cross-bridge treat myosin as a simple linear spring arranged parallel to the contractile filaments. These single-spring models cannot account for the radial force that muscle generates (orthogonal to the long axis of the myofilaments) or the effects of changes in filament lattice spacing. We describe a more complex myosin cross-bridge model that uses multiple springs to replicate myosin's force-generating power stroke and account for the effects of lattice spacing and radial force. The four springs which comprise this model (the 4sXB) correspond to the mechanically relevant portions of myosin's structure. As occurs *in vivo*, the 4sXB's state-transition kinetics and force-production dynamics vary with lattice spacing. Additionally, we describe a simpler two-spring cross-bridge (2sXB) model which produces results similar to those of the 4sXB model. Unlike the 4sXB model, the 2sXB model requires no iterative techniques, making it more computationally efficient. The rate at which both multi-spring cross-bridges bind and generate force decreases as lattice spacing grows. The axial force generated by each cross-bridge as it undergoes a power stroke increases as lattice spacing grows. The radial force that a cross-bridge produces as it undergoes a power stroke varies from expansive to compressive as lattice spacing increases. Importantly, these results mirror those for intact, contracting muscle force production.

## Author Summary

The molecular motor myosin drives the contraction of muscle, but doesn't just produce force in the axis of shortening. Models of muscle contraction have primarily treated myosin as a simple spring oriented parallel to its direction of movement. This assumption does not allow prediction of the relationship between the forces produced and the spacing between contractile filaments or of radial forces, perpendicular to the axis of shortening, all of which are observed during muscle contraction. We develop an alternative model, still computationally efficient enough to be used in simulations of the sarcomere, that incorporates both extensional and torsional (angle dependent, like those found in a watch) springs. Our model captures much of the spacing-dependent kinetics and forces that are missing from single-spring models of the cross-bridge.

## Introduction

Radial forces are the same order of magnitude as axial forces in contracting muscles [1]–[3]. These forces, along with axial force acting in the direction of muscle contraction, depend on myofilament lattice spacing [4], [5]. At the same time, structural information about myosin cross-bridges suggests that they generate force by applying torque to a lever arm [6]–[8]. This lever arm generates the strain accompanying the power stroke via a change in the rest angle at which the lever is attached to S1 region [8], [9]. This change in angle occurs at the converter region, a flexible area in myosin S1 which acts as a torsional spring. These phenomena may be related: the radial forces a cross-bridge creates are results of the lever arm geometry (as suggested by Schoenberg [10]).

**Figure 1:**
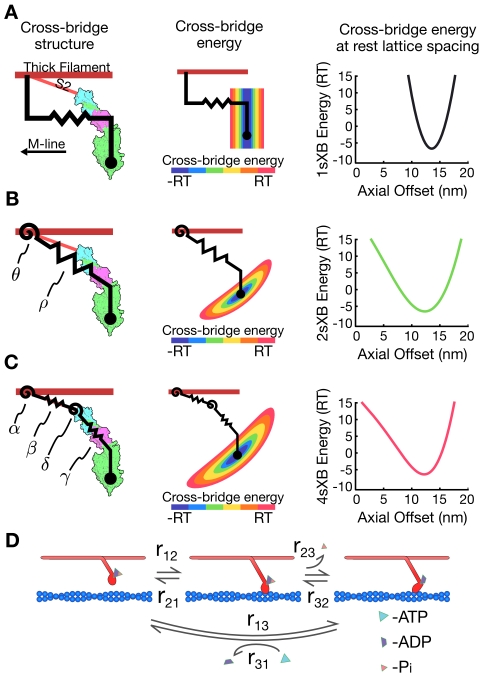
Cross-bridge types and kinetic scheme. (A)–(C) The three cross-bridge models, plotted against a myosin crystal structure for comparison (structure image generated from Gourinath et al. (2003) [40] with PyMol [41]). The energy landscape of each cross-bridge and the free energy at rest lattice spacing are shown adjacent to the cross-bridge schematic. (A) The 1sXB introduced in Huxley (1957) [11]. (B) The 2sXB which uses a torsional/angular spring (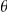) and an extensional spring (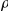). (C) The 4sXB with two torsional and two extensional springs. Of the 4sXB's springs, 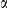 corresponds to the point at which the S2 region rejoins the thick filament backbone, 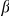 to the S2 region itself, 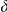 to the area linking the S2 and the light chain domains, and 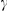 to the light chain domain itself. 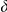 replicates the change in angle accompanying the power stroke by applying torque to the freely moving joint representing the converter domain. (D) The three state kinetic system. The three states represent (1) an unbound state, (2) a pre-power stroke state, and (3) a post-power stroke state. The rate of transition between states 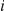 and 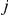 is represented as 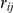. The forward and reverse transition rate constants are functions of energy stored in the cross-bridge.

Existing theoretical and computational models of cross-bridge force generation at the level of the half-sarcomere assume that force is generated by a simple extensional linear spring oriented parallel to the long axis of the myofilaments (Figure 1A). This assumption has persisted from the earliest fundamental models of muscle contraction to more elaborate and spatially explicit models [11]–[15]. These single-spring models yielded insight into the processes that regulate production of force in the direction of contraction, parallel to the long axis of the myofilaments. However, these prior models of muscle contraction have paid less attention to radial forces and the effects of changes in filament lattice spacing. As a result, geometries of the single spring cross-bridge models have changed little while kinetic schemes governing transitions between conformational states have increased in complexity [11], [12], [16], [17]. To analyze the radial forces that occur during muscle contraction, a different cross-bridge geometry is needed: a geometry that produces both forces aligned with and forces orthogonal to the long axis of the myofilaments. A lever arm of several springs can: (1) simulate the deformations a cross-bridge undergoes as it generates force through the power stroke, (2) provide a geometry which is practical for use in cross-bridge models, and (3) account for both axial and radial forces [9].

Here we detail two models of cross-bridges that use multiple springs to replicate the lever arm mechanism and capture its biologically relevant effects (Figure 1B–C). Both models are affected by changes in lattice spacing as well as axial offset from binding sites along the thin filament, and both account for the radial component of force produced during the power stroke. The first model (referred to as the 4sXB model) simulates the cross-bridge as a system of four linearly elastic springs arranged in a geometry based upon the structure of the S1 and S2 regions of myosin II (Figure 1C). Our second model (referred to as the 2sXB model) consists of two linearly elastic springs and provides greater computational efficiency than the 4sXB model while replicating many of the more complex model's behaviors (Figure 1B). A prior two spring cross-bridge model was proposed by Schoenberg (1980), with the S2 arm represented as an extensional spring and the S2-S1 junction as a torsional spring [10], [18]. Both the 4sXB model and the 2sXB model use a three-state model of cross-bridge cycling kinetics, consisting of an unbound state, a low-force pre-power stroke state, and a force-producing post-power stroke state. The kinetics of transition from one state to another in our models are similar to those used previously but are generalized for use in two dimensions; our kinetics calculate transition probabilities using the free energy landscape of the cross-bridges instead of the offset of the cross-bridge head (Figure 1D and Figure S1) [12], [14], [16], [19]. We compare the 4sXB and 2sXB models to a single spring model of the cross-bridge (referred to as the 1sXB model), similar to those used previously. We quantify both the axial and the radial forces of our two cross-bridge models. Additionally, we show how changes in lattice spacing and axial offset affect kinetics and forces in our multiple-spring models.

## Results

The 4sXB and 2sXB models detailed here were developed to discover the consequences of lattice spacing on cross-bridge kinetics and two dimensional force production. Multi-spring cross-bridges introduce a lattice spacing dependence into force production and kinetics, and account for radial forces. As lattice spacing changes, the kinetics and forces of the 4sXB and 2sXB models shift in both magnitude and axial offset.

### At 34 nm 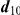, the multi- and single-spring cross-bridges have similar kinetics and energies

**Figure 2:**
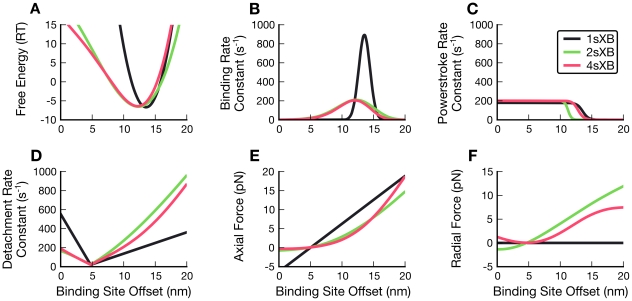
Forces, energy, and kinetics of the 1sXB, 2sXB, and 4sXB models at resting lattice spacing. (A)–(F) show the energy, transition rate constants, and forces of the 1sXB model (black), 2sXB model (green), and 4sXB model (red) at resting lattice spacing. The 1sXB model values shown for comparison are derived from those of Daniel et al. (1998) and Tanner et al. (2007), [12], [14], shifted axially so the resting location of the cross-bridge head in each case is aligned with the resting locations of the 2sXB model and 4sXB model allowing easier comparison. The free energy of the cross-bridges in state two is shown in (A), where the multi-spring cross-bridges' shifts from a purely parabolic trajectory is visible. The explicit two-dimensional thermal forcing of the multi-spring cross-bridge heads in (B) results in binding probabilities that are more distributed than those of the single spring cross-bridge. The rate of power strokes (C) remains least changed between the single and the multi-spring cross-bridge models. The energy-based kinetics of the multi-spring cross-bridges are unable to fully replicate the biased detachment rate of the 1sXB model in (D). (E) and (F) show the 1sXB's sharp discontinuities in axial force and lack of any radial force.

At rest lattice spacing, the free energies and kinetics of the of the single- and multi-spring cross-bridge models are largely similar, as seen in Figure 2 (where the 1sXB values used are calculated as in Figure 10 of Tanner et al. (2007) [14]). These properties share a common base that is intentionally conserved, where possible, between the multiple-spring and single-spring cross-bridges [16]. The free energies of the multi-spring cross-bridges are a result of both extensional springs that are at an angle to the thick filament and torsional springs sensitive to the angle they make with the thick filament. As the multi-spring cross-bridges move in the axial direction, their angles to the thick filament backbone change. This angle dependence skews the free energies of the multi-spring cross-bridges from the symmetric hyperbola of the 1sXB (Figure 2A). The two-dimensional diffusion-based binding probability function that governs the multi-spring cross-bridges (as described in the binding rate calculation section) causes the likely binding areas to occupy a greater range of axial positions than those of the single-spring cross-bridge (Figure 2B) [20], [21]. Multi-spring cross-bridges are thus less likely than the 1sXB model to bind near their rest position, but are more likely to bind than the 1sXB at greater offsets from their rest position. This flattening and spreading of the binding probability function is a result of the extra degrees of freedom of motion in the two-dimensional models. The power stroke rate constants of the multi-spring cross-bridges are the same as those of the single-spring cross-bridge, with energy-dependent terms using the sum of the free energy of every spring comprising a cross-bridge (Figure 2C). The detachment rate constant of the 1sXB explicitly relies on cross-bridge head position as well as energy. This position dependence was removed in adapting the 1sXB model's detachment rate constant for the multi-spring cross-bridges. The detachment rate constant thus loses the intentional asymmetry that the position term provided and retains only the asymmetry created by the spring geometries of the 2sXB and 4sXB models (Figure 2D). The rate of detachment and the other cross-bridge kinetic rate constants remain close to those of the 1sXB, even though the kinetics of the multi-spring cross-bridges are based not on axial position but on the free energy of the cross-bridge in multiple dimensions.

### Axial offsets of most cross-bridge properties decrease as lattice spacing grows

**Figure 3:**
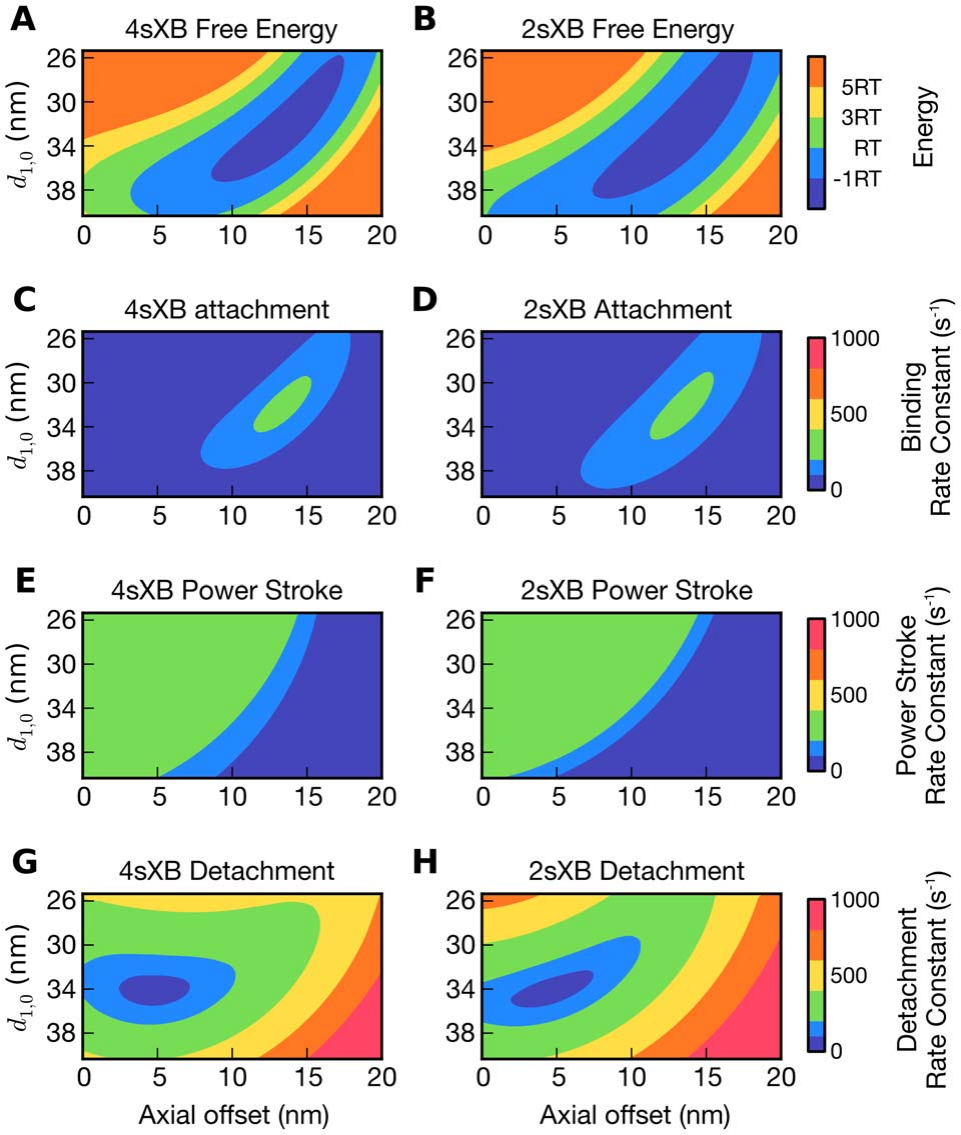
Energy and kinetics of the multi-spring cross-bridge models change with axial offset and lattice spacing. Axial offset is the distance between the current axial location of the cross-bridge tip and the location where the cross-bridge attaches to the thick filament. Lattice spacing (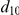) is defined as in Millman (1998) [3], with an offset to account for filament thicknesses so the cross-bridge spans the filaments at a rest lattice spacing of 34 nm. (A)–(H) The properties of the 4sXB model (A, C, E, and G) and the 2sXB model (B, D, F, and H) as they change with binding site offset and lattice spacing. (A) depicts the free energy of the 4sXB model at various lattice spacings, with the head stretched to an axial offset from the thick filament attachment point. The free energy of the 2sXB model is shown in (B). (C) and (D) show 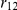, the probability that the 4sXB and 2sXB models will transition from an unbound state to a bound state. (E) and (F) show 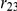, the probability of transition from a pre-power stroke state to a post-power stroke state, for the same cross-bridges, axes, and scales as (C) and (D) show 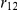. (G) and (H) show 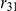, the probability of unbinding from a post-power stroke state. The reverse rate constants, 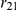, 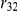, and 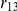 are back-calculated from the forward rate constants.

The axial offset of a cross-bridge property is the axial distance from the point where the cross-bridge attaches to the thick filament to the point where the cross-bridge property reaches an extreme value or inflection point. These axial offsets are depicted in Figure 3 and Figure S2 where, for example, the axial offset of the 2sXB attachment rate constant at 34 nm 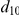 is approximately 12 nm. As lattice spacing increases, the axial offsets of most multi-spring cross-bridge kinetic rates and free energies grows smaller. This relationship is shown in Figures 3A and B and Figure S2 A and B, where the axial offset of the 4sXB or 2sXB model's lowest energy point is more than 3 nm greater at a lattice spacing of 32 nm 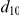 than at a lattice spacing of 38 nm 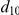. The positions where cross-bridges are most likely to bind shift to smaller axial offsets at larger lattice spacings, decreasing how extended a cross-bridge is likely to be upon binding (Figures 3C–D and Figure S2C–D). Similarly, as lattice spacing increases, decreases in the axial offset of the power stroke rate constant inflection point cause the size of the power stroke to change with lattice spacing (Figures 3E–F and Figure S2E–F). The 4sXB model's rate of detachment is the only cross-bridge property whose axial offset is predominately invariant with changes in lattice spacing (Figure 3G and Figure S2G). This exception is explained by the largely radially aligned post-power stroke orientation of 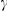, the 4sXB model's final spring. Combined, these effects reduce the axial force a cross-bridge generates at larger lattice spacings with implications for the sarcomere length dependence of force production and relaxation. These multi-spring cross-bridge models are the first to be capable of reproducing these lattice spacing dependent effects on force production and kinetics.

### Probability of a cross-bridge being bound decreases as lattice spacing diverges from rest

The number of cross-bridges in a force generating state depends on lattice spacing. At any axial location, as lattice spacing diverges from its 34 nm 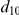 rest value, the rate of attachment decreases while the rate of detachment increases (Figure 3C–D and 3G–H). These kinetic rate constants change with lattice spacing because they depend on the difference in free energy between the unbound state and the pre- or post-power stroke state, a difference which increases with lattice spacing. This increase in energy makes a cross-bridge increasingly likely to transition to the unbound state and remain there (Figure 3C–D and 3G–H). An example of the decrease in the likelihood of a cross-bridge remaining bound can be seen in the 4sXB model, where the slowest rate of detachment is 20/sec at a lattice spacing of 34 nm 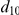 but rises to 260/sec at 38 nm 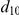 (Figure 3G). As a result of these changes, individual cross-bridges spend less time in a bound state and are less likely to generate force as lattice spacing diverges from its rest value.

### Forces at a given axial offset increase with lattice spacing

**Figure 4:**
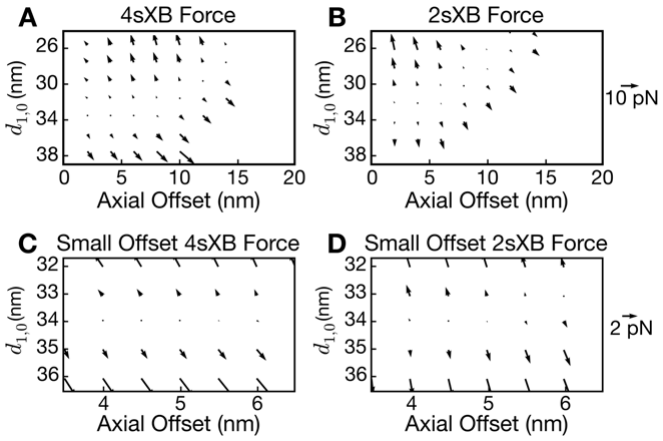
Overview and detail of the forces exerted by the 2sXB and 4sXB models in the post-power stroke state. (A)–(D) show the post-power stroke forces exerted by the 4sXB and the 2sXB models as vector fields of reaction forces. The reaction force is that necessary to retain the cross-bridge head in a given location, thus the vectors for a compressed cross-bridge orient upwards and those for an extended cross-bridge orient downwards. Positions in which the cross-bridge is unlikely to generate force are omitted; these unlikely locations are determined by the sum of 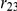 and the inverse of 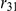. (A) and (B) show overviews of the forces exerted, respectively, by the 4sXB model and the 2sXB model over lattice spacings and axial offsets that vary as in Figure 2. The forces exerted by the two cross-bridges have radial components which frequently equal or exceed their axial components. A more detailed view of the region surrounding the rest position of the cross-bridges is shown in (C) and (D), where the large radial components of the cross-bridge forces, particularly for the 2sXB model, is especially evident.

**Figure 5:**
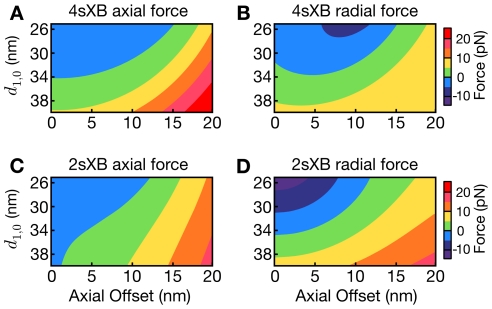
Axial and radial post-power stroke forces as separate components. (A)–(D) show, separated, the axial and radial components of the forces produced by the 4sXB and the 2sXB models in the post-power stroke state.

The axial and radial forces at a given axial offset correlate with lattice spacing (Figures 4 and 5). When lattice spacing is compressed, more expansive radial forces and smaller axial forces are produced. When lattice spacing is expanded, more compressive radial forces and larger axial forces are produced. An example of increased forces with increased lattice spacing is seen in the 4sXB model which, at a 10 nm axial offset, produces half the radial and half the axial force at 35 nm 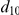 as it does at 38 nm 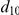 (Figure 5A–B). Similarly with the 2sXB model at a 12 nm axial offset, a lattice spacing of 35 nm 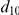 produces two thirds of the axial and radial forces as does a lattice spacing of 38 nm 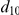 (Figure 5C–D). At large lattice spacings, this greater force per cross-bridge competes with the decreased probability a cross-bridge will bind and generate force, an interaction that requires a model of the half-sarcomere using multi-spring cross-bridges to fully evaluate [22].

The force landscapes of Figure 5 also show that no lattice spacing is free of radial force at all axial offsets. The radial force produced by a cross-bridge, even at rest lattice spacing, increases in magnitude as the cross-bridge tip moves away from its unstrained axial offset.

### Step size varies with lattice spacing

**Figure 6:**
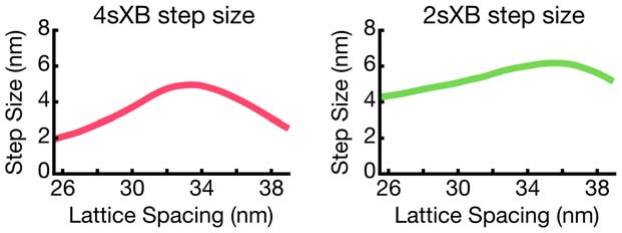
Changes in step size with lattice spacing. Step size varies as lattice spacing diverges from its rest value. Step size is defined as the change in the rest axial offset between the pre- and post-power stroke states. The step size of the 4sXB model and 2sXB model produce different absolute step sizes as lattice spacing change. However, both models exhibit a local maximum step size at a specific lattice spacing with a decreasing step size as lattice spacing diverges from that point.

The step size of both multi-spring models varies with lattice spacing (Figure 6). We define step size at a given lattice spacing as the axial distance between the pre- and post-power stroke positions of the myosin head. Put another way, step size at one lattice spacing is the distance from the axial offset with the lowest free energy in the pre-power stroke state, to the axial offset with the least amount of energy in the post-power stroke state. Both models have a peak step size at a relatively uncompressed lattice spacing, with decreasing step size as lattice spacing diverges from that value. The 4sXB model has a maximum step size of 5.0nm near 34nm lattice spacing and the 2sXB model has a maximum step size of 6.1nm near 36nm lattice spacing.

### Radial forces are of the same order of magnitude as axial forces

The radial and axial components of force, produced by a 4sXB model or 2sXB model moved from its rest position to an axial offset, are of the same order of magnitude (Figures 2E–F and 4A–D). The values of the axial and radial forces produced by the multiple-spring cross-bridge models at rest lattice spacing are compared to those produced by the single-spring cross-bridge model in Figure 2E–F. The relative values of the radial and axial forces are visualized as the angles of the force vectors in Figure 4A–D. Axial locations and lattice spacings with balanced axial and radial forces produce force vectors which are neither vertical nor horizontal, but in some intermediate orientation. Most axial and radial offsets are populated by such vectors, particularly regions a cross-bridge would be most likely to occupy (unlikely regions are not shown in the vector plots). The few regions dominated by one force, notably some small offset positions in the 2sXB model (Figure 4D), are dominated by radial forces. This presence of large radial forces suggests that, in all but the least strained locations at the smallest axial offsets, radial forces will be present in magnitudes comparable to those of axial forces.

## Discussion

Our multi-spring cross-bridge models show how myofilament lattice spacing influences cross-bridge properties, from axial and radial forces to kinetics and step size. The 4sXB and 2sXB models show two key features that differ significantly from prior models: (1) the inclusion of torsional springs and lever-arm mechanisms reveals a dependence of step size on lattice spacing and (2) this lever-arm mechanism produces radial forces and axial forces of the same magnitude, a ratio similar to that observed experimentally [1], [2], [23]. The dependencies of step size, force production, and kinetics on lattice spacing help explain measured changes in force generation with changes in lattice spacing [3].

### Force generated by a multi-spring cross-bridge depends on lattice spacing

The lattice spacing of the filaments around an attached multi-spring cross-bridge determine the energy landscape of the cross-bridge and thus the force it can generate. The forces and strains a cross-bridge produces at most axial offsets grow more positive as lattice spacing increases (Figure 4E–H). While this increased cross-bridge strain translates into greater axial and radial force per post-power stroke cross-bridge, the probability that these cross-bridges will bind decreases as lattice spacing increases (Figure 3C–D). The decrease in attachment rate constants at extreme lattice spacings, while power stroke rate constants remain unchanged (Figure 3E–F), suggests lattice spacing influences muscle fiber force generation by altering the rate of cross-bridge attachment rather than the power stroke rate [22]. Spatially explicit effects in the compliant sarcomere, such as cross-bridge induced realignment of binding sites, may act to balance the decreased binding and increased detachment at larger lattice spacings.

### The 2sXB model approximates the 4sXB model

The energies, kinetics, and forces generated by the 2sXB model are subject to the same governing trends as those of the 4sXB model, and can be made similar by deliberate parameter choice (Table 1 and Figures 2, 3, 4, and 5). That the 2sXB model can replicate the results of the 4sXB model indicates two things: first, the 2sXB can be used in place of the 4sXB in larger simulations, enabling work that would otherwise require prohibitive resources, and second, a feature shared between our two models is responsible for the interesting properties of our simulations, the use of a lever arm which undergoes an angle change to generate force. While the energies, binding rate constants, and power stroke rate constants of the multi-spring cross-bridges are almost identical, there are some smaller differences between the two models. The rate constant of detachment is rotated by approximately 20 between the two systems due to differences in the way the post-power stroke position is achieved (Figure 3). The 4sXB model and the 2sXB model generate somewhat different forces; the axial force produced by each model increases with lattice spacing, but that produced by the 4sXB does so more steeply (Figure 5A, C). In a reversal of this pattern, the 2sXB model's radial force is more dependent on lattice spacing (Figure 5B, D). In each of these cases, the forces generated by both multi-spring cross-bridges are subject to the same trend. The close agreement between the forces and other properties of the two cross-bridge representations supports the position that the key feature of our multi-spring models is the use of a lever arm to generate force, rather than a factor unique to the 4sXB model, such as the simulation of interaction between the lever arm and the S2 domain. Substituting the 2sXB model for the 4sXB model reduces the runtime of a simulation by two orders of magnitude and puts multi-spring cross-bridge simulations of the half-sarcomere within reach.

**Table 1:** Model parameters and their sources.

Model	Spring	Rest value	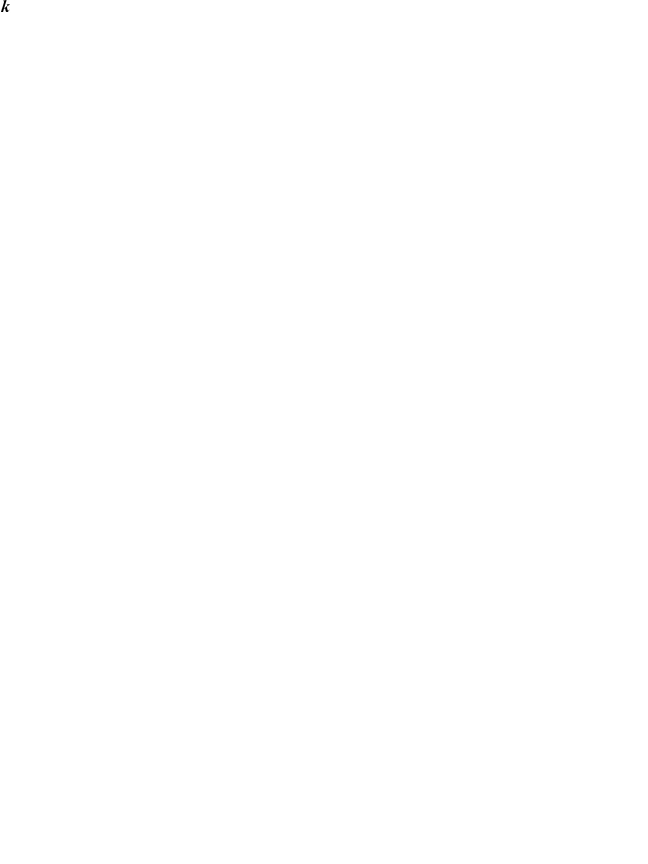	Source
4sXB	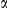	40 	100 pN/rad	[42]
	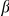	10.5 nm	10 pN/nm	[42]
	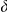	125 	40 pN/rad	[37]
	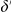	70 	40 pN/rad	[37]
	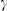	9.6 nm	5 pN/nm	[36]
2sXB	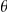	47 	40 pN/rad	See caption
	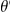	73 	40 pN/rad	See caption
	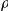	20 nm	2 pN/nm	See caption
	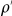	16 nm	2 pN/nm	See caption
1sXB	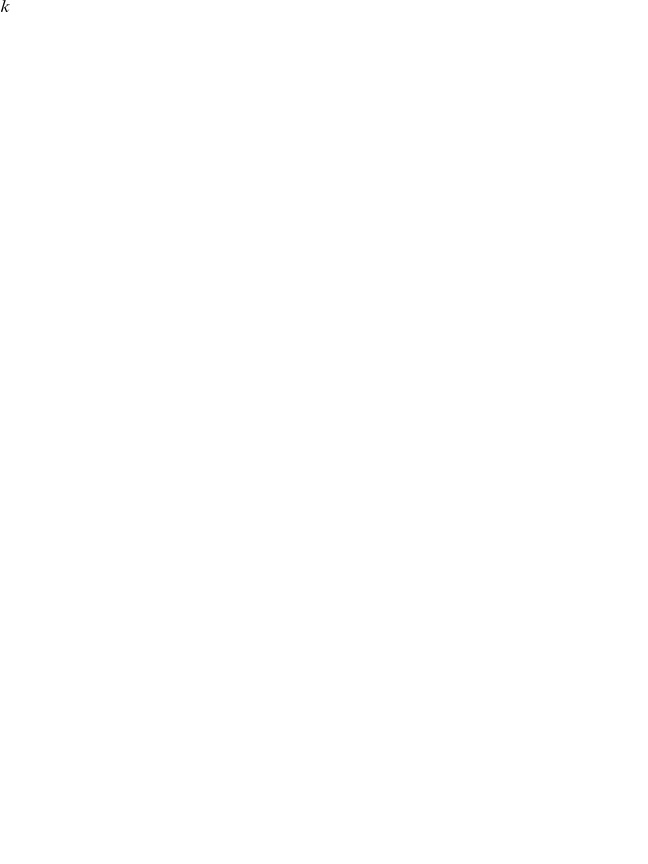	5 nm	5 pN/rad	[14]
	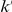	0 nm	5 pN/rad	[14]

Prime values, such as 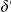, represent post-power stroke state values. From Liu et al. (2006) [42], which used insect flight muscle, the most frequently occurring thick filament to S2 angle range is 51–60. We assume that this range is being distorted by the compressive radial force being generated by the rigor cross-bridges in the swollen lattice spacings that Liu et al. used. As such, we choose a rest angle for 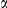 at the low end of the still common range of 50 to 40. We do not change this angle between states one, two and three. In Taylor et al. (1999) [37] (clearly explained in [43]) the angle between the LCD and the thick filament's axial axis goes from 125 to 70 with the power stroke. The LCD rest length generated by measurements made of structure 1DFK from Houdusse et al. (2000) [36]. The rest values of the 2sXB model's springs are determined by those of the 4sXB model; they are calculated so that the rest position of the 2sXB's head is the same as the rest position of the 4sXB's head. The spring constant, 
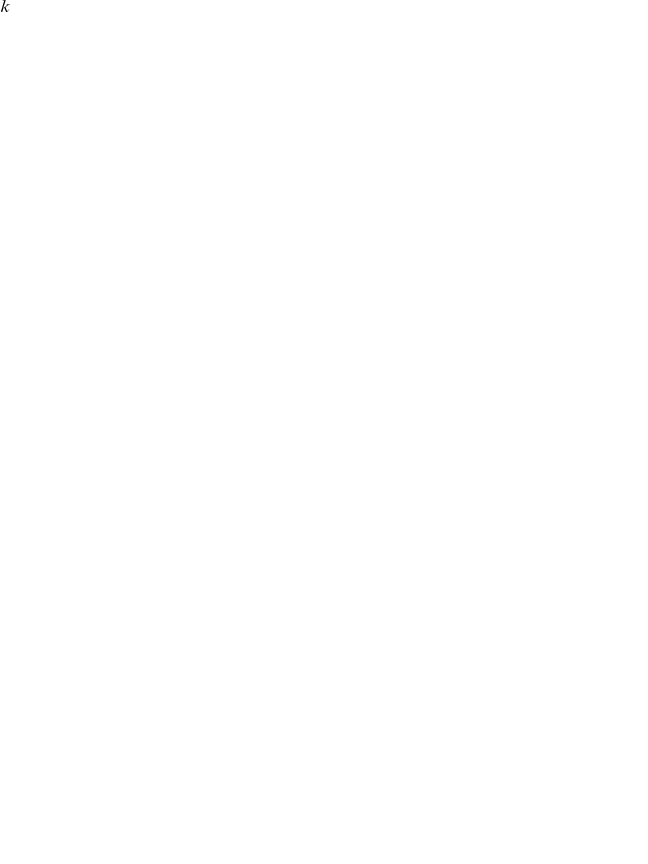
, for the angular spring responsible for each cross-bridge's power stroke is determined by the change in angle over the power stroke and the energy liberated by the hydrolysis of ATP [14]. Additional spring constants are chosen to be consistent with previous work, and to provide sufficient flexibility to enable diffusion. The parameters of the single spring cross-bridge, used for comparison, are taken from [14].

### Cross-bridge step size depends on lattice spacing, influences shortening velocity

The geometries of the multi-spring models require a change in step size accompany a change in lattice spacing. This is because, while the length of the lever arm changes as lattice spacing varies, the pre- and post-power stroke angles do not. Step size varies more in the 4sXB model as the 4sXB model's spring configuration causes the pre- and post-power stroke free energies to differ more than in the 2sXB model. As the detachment rate constant is a product of the post-power stroke free energy, the greater rotation in the 4sXB's post-power stroke free energy, relative to that of the 2sXB model, can be seen in Figure 3 G–H. Experimental measurements of step size vary, and it has been postulated that this is due to more than experimental error, but to our knowledge these results are the first prediction of a step size that varies with lattice spacing [24]. Experimental confirmation of these predictions is not possible with current literature: existing *in vivo* measurements of step size are from isolated myosin preparations which are unable to simulate a change in muscle lattice spacing [25], [26].

While our single cross-bridge models lack the predictive power of a multi-filament model, the dependence of step size on lattice spacing offers insight into unloaded shortening velocity. Maximum unloaded shortening velocity is commonly interpreted as a function of both myosin's step size and drag from attached post-power stroke cross-bridges [27]. A decrease in unloaded shortening velocity is observed when lattice spacing is compressed via dextran [28], [29]. This slower unloaded shortening is supported by the multi-spring models: their step size exhibits a similar decrease as lattice spacing shrinks (Figure 6). However, a moderate increase in the rate of detachment at highly compressed lattice spacings, seen in Figure 3 G–H, may balance smaller steps sizes. This increased detachment rate is due to the greater post-power stroke strain that is present with greater radial displacement of the cross-bridge. Changes in modeled detachment rates and step size are both likely to be needed, along with changes in filament overlap, to explain the complicated dependence of unloaded shortening velocity on sarcomere length [30].

### Large radial component of forces may influence lattice spacing in multi-filament models

The 4sXB and the 2sXB produce radial forces of the same order of magnitude as the axial forces generated by a cross-bridge. These forces range between 10% and 50% of the axial force at the least strained axial and radial offsets where a cross-bridge is most likely to enter the post-power stroke state (Figure 4). Muscle fibers display these radial forces by resisting width changes as osmotic pressure is applied [1]. Direct measurement of lattice spacing by X-ray diffraction has confirmed fiber width estimates of radial force [31]. Checchi et al. (1990) [2] observed large radial forces by examining lattice spacing during redevelopment of tension following length changes. A spatially explicit model, even one using multiple thick and thin filaments arranged in a lattice, is insensitive to lattice spacing if it uses a version of the 1sXB model. Embedding multi-spring cross-bridges in a multi-filament model allows the simulation of radial force regulation in a lattice of thick and thin filaments. The inclusion of radial forces in a multi-filament model permits examination of previously unavailable kinds of cooperativity, ones where radial force can be transmitted through the backbone lattice to affect the kinetics of other cross-bridges. Radial force is a potential regulator of lattice spacing and of 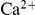 sensitivity as lattice spacing and sarcomere length vary [3]. A multi-filament model using the 4sXB or 2sXB can simulate the interaction of radial force generated by a cross-bridge with radial forces provided by other mechanisms, e.g. titin or electrostatic repulsion [3], [22], [32]. Thus multi-spring cross-bridges make it possible to evaluate the influence of these radial forces, posited to be regulators of lattice spacing, and processes which may depend on lattice spacing or myosin head to thin filament distance, such as the Frank-Starling mechanism; something not possible with a 1sXB model [33].

In future studies, these models will permit the investigation of radial forces and lattice spacing in multi-filament models, and will allow us to examine disease states that alter myosin compliance. The inclusion of radial forces and lattice spacing in half-sarcomere models will illuminate regulatory mechanisms of shortening velocity and length-dependent axial force generation. Other efforts may use existing studies of how disease-related mutations alter myosin compliance to produce disease state mimicking cross-bridge models [34]. Multi-filament simulations using these altered cross-bridge models have the potential to explain how symptoms of disease states such as hypertrophic cardiomyopathy arise from myosin-level changes.

## Models

Our two cross-bridge models, the 4sXB model and the 2sXB model (Figure 1B–C), are designed to capture a range of mechanical behaviors observed or posited by prior work, namely radial force generation and the effects of lattice spacing on cross-bridge binding and force generation. Both cross-bridge models are an arrangement of linearly elastic torsional (angular or watch-like) or Hookean (extensional) springs.

### Geometry

#### Spring configurations

**Figure 7:**
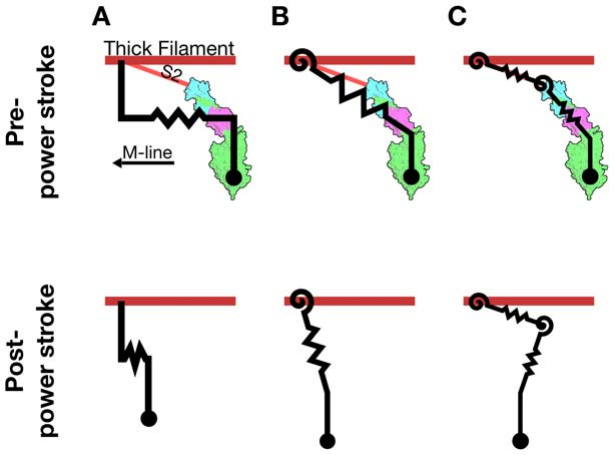
Changes in cross-bridge resting geometry with the power stroke. (A)–(C) show, schematically, the change in the rest lengths and angles of the single and multi-spring cross-bridges. The rest length and angle of the 2sXB's extensional and torsional springs are set, in both the pre- and post-power stroke states so as to match the tip position of the 2sXB in each condition to that of the 4sXB (in Table 1). The change in the unstressed radial distance from the thick filament to the tip of the multi-spring cross-bridges that occurs with the power stroke is particularly visible in (B) and (C) when compared to the single spring cross-bridge (A). The effects of the universal joint attaching the springs of the 4sXB and the 2sXB to the globular domain, and the globular domain's own fixed angle to the thin filament, are shown by the continued radial orientation of the globular domain after the power stroke occurs.

To enable comparison with previous cross-bridge models, we implement a one-dimensional model in addition to our multi-spring models. This one-dimensional model uses a linearly elastic spring oriented parallel to the long axis of the thick filament (Figures 1A and 7A). The resulting cross-bridge forces are restricted to the direction of shortening, that is, axially oriented. The one-dimensional 1sXB model cannot yield radial forces. Moreover, this model's geometry is unable to account for changes in kinetics or forces at varying lattice spacing. This reference model is identical to those used in recent spatially-explicit computational analyses [12]–[14].

The 4sXB model uses two extensional and two torsional springs to represent the myosin head (Figures 1C and 7C). This arrangement of four springs corresponds closely to regions of the cross-bridge believed to regulate and respond to strain or deformation [9], [35]. In particular, the four springs correspond to the point where the S2 region attaches to the rod, the S2 region, the point where the S2 region attaches to the light chain domain (LCD), and the LCD. These points are labeled 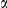, 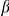, 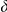, and 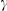, respectively (Figure 1C). Rest values, stiffnesses, and their sources are detailed in Table 1.

The rest angle of 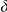 decreases to simulate the transition from a pre-power stroke to a post-power stroke state (Figure 7C). This method of force generation acts in two dimensions and thus allows lattice spacing to influence forces and state transition rates. In the 4sXB, a change in the rest angle of 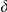 mimics myosin's lever-arm mechanism of force generation [9], [36]. As the extensional spring 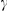 does not bend and the angle at which the globular domain attaches to actin remains unchanged, applying torque at one end of 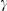 is equivalent to applying the opposite torque at the opposite end. Thus a change in the rest angle of 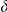 produces a torque equivalent to that which the converter domain applies to the LCD during the power stroke.

The 2sXB model is a simplification of the 4sXB model, using one extensional spring (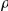) and one torsional spring (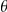) to represent the myosin head (Figures 1B and 7B). The 2sXB treats the power stroke as a change in the rest angle of 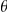 (Figure 7B); like the 4sXB, the 2sXB generates force by applying torque to a lever arm. The parameters of the 2sXB are set so that the pre- and post-power stroke tip location and kinetics of the 2sXB match those of the 4sXB model. In addition to the change in the rest angle of 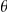 during the power stroke, we adjust the length of 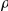 so that the base-to-tip distance of the 2sXB in both the pre- and post-power stroke states is equal to the same measurement in the 4sXB model. The result is computationally simpler than the 4sXB model, but retains the 4sXB's two-dimensional behavior.

The 2sXB presented here is contrasted to an alternative geometry used by Schoenberg [10], [18], where an extensional spring representing the S2 domain is joined, via a torsional spring, to a rigid rod representing the S1 domain. The use of this alternative geometry requires the position of the torsional spring linking the S1 and S2 domains be found through iterative solution methods whenever the cross-bridge tip position changes. This use of iterative solution methods is similar to that required by our 4sXB and imposes similarly large computational requirements when incorporated into larger spatially explicit models. Additionally, this alternative geometry restrains the cross-bridge tip to an area within one S1 length of the line in which the S2 segment is set.

Parameters used in both cross-bridge models are derived, where possible, from existing experimental data, described below. Each extensional spring (one in the 1sXB model, two in the 4sXB model and one in the 2sXB model) has a rest length and a spring constant, while each torsional spring (two in the 4sXB model and one in the 2sXB model) has a rest angle and a spring constant. The lengths and angles of the springs used for the 4sXB are based on tomographic reconstructions of *in vivo* S2 lengths and x-ray crystallographic reconstructions of the S1 fragment [6], [37]. The rest length and angle of the springs used in the 2sXB model are set so that the tips of both the 2sXB's and 4sXB's simulated myosin heads are in the same location before and after the power stroke.

#### Calculation of lattice spacing

The multi-spring models use an internal representation of lattice spacing that is analogous to the *in vivo* distance from the surface of a thick filament to the surface of an adjacent thin filament. However, since this surface-to-surface lattice spacing (ssLS) is not commonly reported, we present lattice spacing as the 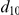 measurement used in x-ray diffraction studies of muscle [3]. The 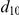 value is the distance between the centers of mass of adjacent thick filaments. We calculate the 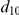 value that corresponds to a given ssLS using both the geometry of the cross-bridge and the lattice spacing at which the cross-bridge generates the least radial force. Specifically, 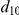 is found from 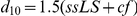 [3]. The correction factor (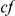) compensates for the filament radii: the difference between the ssLS surface-based measurement and the 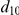 center-of-mass-based measurement. The correction factor offset also sets the relationship between ssLS and 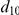 so that, at rest lattice spacing, the post-power stroke cross-bridge generates neither compressive nor tensile radial force. This offset becomes 6.90 nm when the rest 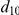 spacing is 34 nm [23]. The ssLS that correspond to the 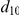 spacings of interest are then calculated and define the window of lattice spacings we examine [3]. Thus the lattice spacing within the model is bound by experimental lattice spacings and is a function of both the geometry of the actomyosin lattice and the lattice spacing at which radial forces are minimized.

#### Displacement and force generation

Each cross-bridge undergoes a distortion as myosin hydrolyzes ATP to 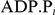; this distortion is the basis of the power stroke [12], [14], [16]. The energy liberated by the hydrolysis of ATP drives force generation by inducing strain in the cross-bridge, appearing as a change in the cross-bridge rest length [25]. For the 1sXB model, this distortion is represented as a change in the rest length of the cross-bridge's only spring (Figure 7A). The 4sXB and 2sXB models use a process which adheres more closely to the *in vivo* lever-arm mechanism; they represent the power stroke as a change in the rest angle of a torsional spring (Figures 7B,C) [38]. The force generated by this process has both axial and radial components. The axial component of the force vector is the portion that lies along the long axes of the thick and thin filaments. The radial component of this vector lies perpendicular to the thick and thin filaments, orthogonal to the axial component. The relative values of the post-power stroke axial and radial forces are determined by the construction of the cross-bridge (number of springs and their geometry), and the displacement of the cross-bridge tip from its rest position.

#### Calculation of spring lengths and angles

To calculate the force and energy a cross-bridge produces and stores as its tip is displaced, we need to know the lengths and angles of the springs that constitute the cross-bridge. When the 1sXB model is placed under strain, the tip of its myosin head moves to a new axial offset. Finding the length of the 1sXB model's spring is simple, as it must span the complete distance from the cross-bridge tip to the thick filament attachment site. Finding the lengths and angles of springs in the 4sXB and 2sXB models is a two-dimensional problem; they must account for both the axial and radial distance from cross-bridge tip to cross-bridge base. The values of the 2sXB model's springs are determined analytically, as both spring values are set by the choice of a head location. The 2sXB model's spring values, 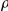 and 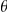, are given by 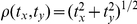 and 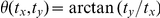, for a cross-bridge tip location of 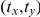 (Figure 1B). The 4sXB model has a greater number of springs and thus another point whose location must be defined: 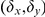, the S2/LCD linking point where the angular spring 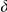 is located (Figure 1C). The coordinates of the 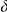 spring cannot be analytically determined, they must be found through iterative optimization. We use a modification of Powell's “dog-leg” method (from the SciPy computational package [39]) to locate the 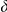 spring such that the 4sXB model is at its lowest energy state for the current cross-bridge tip position. Once 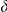
's location is known, its angle, the angle of 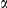 and the lengths of 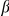 and 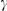 are determined analytically. The angles and lengths for a given tip location 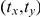 and 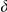 location 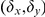 are given by:
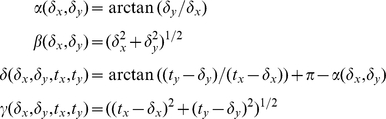



### Kinetics

To describe the kinetics we use a simplified three-state model of the cross-bridge cycle originally described by Pate and Cooke (1989) [16] and modified by Tanner et al. (2007) [14]. This relatively simple scheme directly links the cross-bridge's kinetics and mechanics; the three kinetic states are directly comparable to the myosin configurations described in Houdusse (2000) [36]. The kinetic rates are independent of the number of springs used in a model cross-bridge, allowing the 4sXB and the 2sXB models to use the same system. The three states represented in the kinetic scheme are (1) an unbound state: Myosin-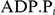 (2) a loosely-bound state:Actin-Myosin-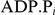 and (3) a force-generating post-power stroke state: Actin-Myosin-ADP (Figure 1D). These kinetics replicate those of a generic cross-bridge, and are aimed at reproducing properties shared between cardiac, skeletal, and insect myosin types.

The kinetics of both the 4sXB and the 2sXB models are strain dependent and are essentially transforms of the free energy landscapes experienced by the cross-bridges in their different states. These free energies are a function of the distortion necessary to move the point representing the simulated myosin head's tip to the proposed binding site. Examples of these free energy landscapes are shown in Figure 3A and B, with cuts through them at the rest lattice spacing visible in Figure 2A. As the free energies of the cross-bridges are functions of their spring rest values and stiffnesses, changing the geometry and stiffness of the springs used by the model also changes the kinetics of the model.

The binding probabilities of both the 4sXB and the 2sXB models are determined by Monte-Carlo simulations of their diffusion as a result of being perturbed by Boltzmann-derived energy distributions [21]. After a new head location is found, a binding probability is calculated which decreases exponentially with distance from the potential binding site. This probability is tested against a random number from a uniform distribution to determine if binding occurs in our chosen time step of 1 ms.

#### Free energy in each state

The total free energy liberated by the hydrolysis of the gamma 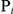 of ATP and available to the myosin head over the course of a cross-bridge cycle (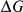) depends on both the standard free energy of ATP hydrolysis (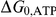) and the concentrations of ATP, ADP and 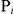. The free energy available to the cross-bridge over its cycle is given by 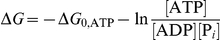. The free energy in the unbound state serves as a reference for the other states and is set to 0. As the unbound cross-bridge supports no strain, its free energy (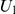) remains at 0 for all axial offsets and lattice spacings. Only a portion of the liberated free energy is available to the cross-bridge in a given state. The limits on available 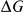 are included in the free energy of each state as an efficiency factor, as in Tanner et al. (2007) [14], [16]. The weakly bound state's efficiency is 28%, represented with 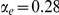, and the strongly bound state's efficiency is 68%, represented with 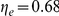. The free energy of a cross-bridge in each state also depends on the strain the cross-bridge experiences from distortion upon binding. Thus the free energy of the cross-bridge in state 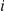 (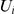) is a linear combination of the strain-dependent and phosphate-dependent energy of the cross-bridge. The free energies of the 4sXB system are:
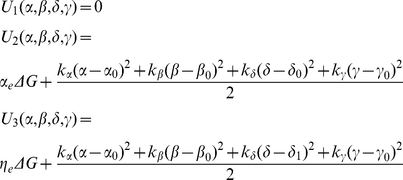
(1)The free energies of the 2sXB system are:
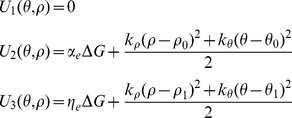
(2)


#### Binding rate calculation

Our binding algorithm follows Tanner et al. (2007) [14] but differs in two key ways: (1) we split binding to the thin filament into two steps, and (2) our diffusion step works with any number of springs. Previous models treated binding rate constants as an exponential function of the distance between a tethered diffusing spring and an available binding site [12], [14]. We produce binding rate constants in the same fashion, but in adapting them for multi-spring cross-bridges, split the process into two steps: diffusion of the myosin head to a new location, followed by calculating binding probability at the new location. The energy of a single spring undergoing thermally forced diffusion is taken from a Boltzmann distribution of possible energies [20], [25]. With a single-spring cross-bridge, the cross-bridge tip offset is easily found from the energy of the spring. A thermally forced multi-spring cross-bridge likewise takes the energy for each of its constituent springs from a Boltzmann distribution of energies, but tip location must be separately calculated (see the Geometry section above). It is this separation of the calculation of cross-bridge tip location from binding probability that splits our binding rate calculation into two steps.

In the diffusion step, each spring is offset from rest with an energy taken from the probability density function: 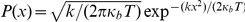 where 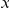 is the offset, 
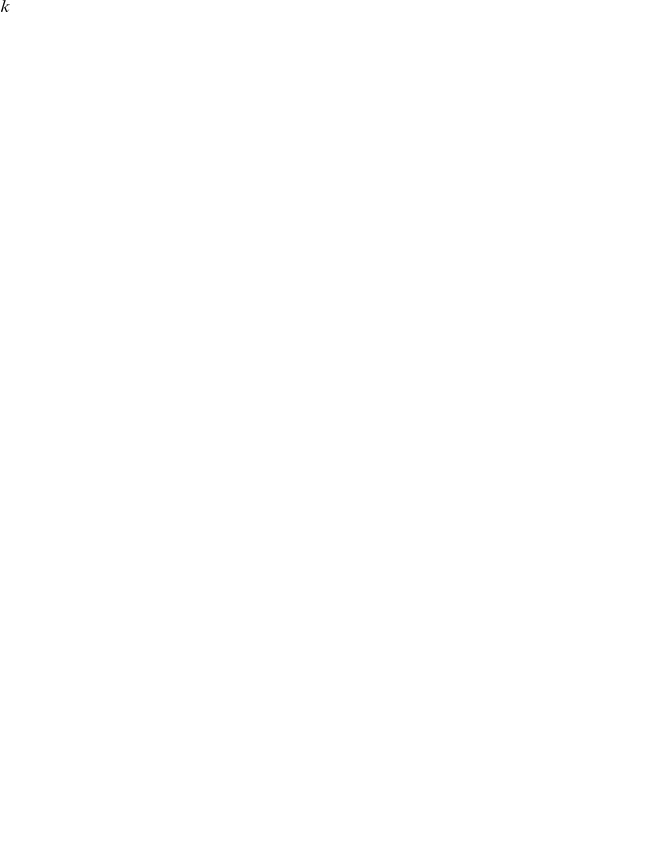
 is the spring constant of the particular spring, 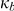 is the Boltzmann constant, and 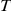 is the system's temperature in Kelvin [21], [25]. The new spring values are used to update the location of the cross-bridge tip, which is used to calculate the post-diffusion distance, 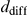, from the tip to the binding site of interest. As in previous models, the probability that the cross-bridge will bind to a given binding site decreases exponentially as 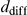 increases. Thus the probability a cross-bridge will bind to an available site is given by 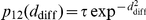, where 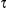 is a scale factor with a value of 12 for the 4sXB and 72 for the 2sXB, chosen to provide attachment rates consistent between the multiple spring cross-bridge models. Attachment occurs when 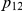 is greater, on a given 1 ms time step, than a random number chosen from a uniform distribution in the domain 0 to 1 [14]. This process is sufficient to determine if a cross-bridge in simulation binds in a given time step, but binding rate constants, as used in Figures 2 and 3 are calculated with an ensemble of cross-bridges. Thus, for an ensemble of size 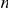:
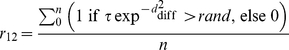
(3)This two step system, with diffusion followed by a chance of attachment, is used for both the 4sXB and 2sXB models with only a change in the number of thermally forced springs and the scaling factor 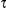.

#### Power stroke and detachment rates

The power stroke and detachment rates are adaptations of prior models [14], [16]. Unlike with binding, the power stroke and detachment rate constants explicitly depend on the free energy of the cross-bridge. This free energy is calculated from equations 1 and 2, with the tip co-located at the relevant binding site. In the case of the 4sXB, each calculation of a transition rate constant requires that the location of the converter domain be optimized to relax the cross-bridge into its lowest energy state. Of note, the unbound cross-bridge supports no strain and so 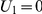. These rate constants are insensitive to the number of springs comprising each cross-bridge and function similarly in one- and two-dimensional models. Both the power stroke rate constant (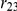) and the detachment rate constant (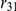) depend on the differences in free energy between the current state and the one being considered for transition. This dependence on the difference in free energies means transitions are more likely when they are energetically favorable and less likely in other circumstances, a natural scheme based in the geometry of the cross-bridges. The particular rate constants for both the 4sXB and the 2sXB models are:
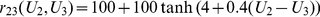
(4)

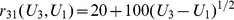
(5)


#### Calculation of reverse rates

The reverse transition rate constant from state 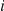 to state 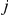 is given by the thermodynamically balancing formula:
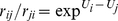
(6)where 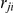 is the forward rate constant and 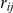 is the reverse rate constant [12], [14], [16]. The transition from a pre-power stroke state to an unbound state requires the reverse transition again be treated as a fraction of an ensemble of transition opportunities, using Equation 3 to provide 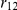. The remaining forward transition rate constants, 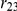 and 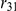, are calculated from equations 4 and 5, while all free energies are provided by equations 1 and 2.

## Supporting Information

Figure S1 Model simulation protocol. The model simulation process, as described throughout the paper, is displayed as a state diagram. Entering the diagram at “Start”, the states and actions which change those states are depicted for a single cross-bridge.
(0.06 MB PDF)

Figure S2 Cross-bridge free energy and kinetics at multiple lattice spacings. (A)–(B) show the free energies of the 4sXB and the 2sXB models at lattice spacings between 30 and 38 nm. (C)–(H) show the kinetic rate constants of the 4sXB and the 2sXB models at lattice spacings between 30 and 38 nm. Each rate is a section taken from the corresponding display in Figure 3.
(0.24 MB PDF)
