# Supplementary material for: Axial and Radial Forces of Cross-Bridges Depend on Lattice Spacing
Source: PLoS Comput Biol. 2010 Dec 2;6(12):e1001018. doi: 10.1371/journal.pcbi.1001018 (PMC2996315; doi:10.1371/journal.pcbi.1001018)
Supplement: Figure S1 — Model simulation protocol. The model simulation process, as described throughout the paper, is displayed as a state diagram. Entering the diagram at “Start”, the states and actions which change those states are depicted for a single cross-bridge. (0.06 MB PDF) [file pcbi.1001018.s001.pdf]

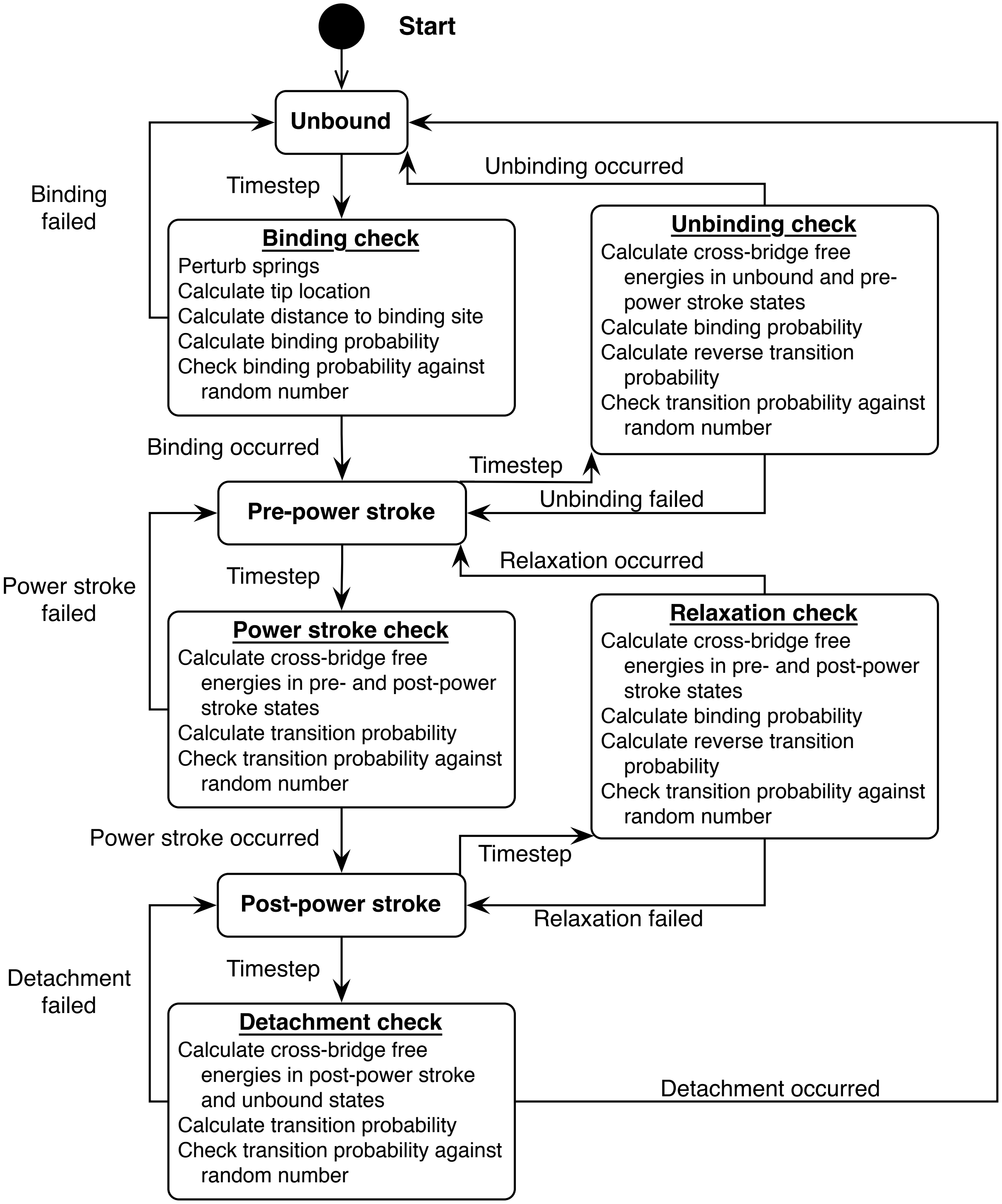

Figure S1: Model simulation protocol The model simulation process, as described throughout the paper, is displayed as a state diagram. Entering the diagram at "Start", the states and actions which change those states are depicted for a single cross-bridge.
