# Supplementary material for: Axial and Radial Forces of Cross-Bridges Depend on Lattice Spacing
Source: PLoS Comput Biol. 2010 Dec 2;6(12):e1001018. doi: 10.1371/journal.pcbi.1001018 (PMC2996315; doi:10.1371/journal.pcbi.1001018)
Supplement: Figure S2 — Cross-bridge free energy and kinetics at multiple lattice spacings. (A)–(B) show the free energies of the 4sXB and the 2sXB models at lattice spacings between 30 and 38 nm. (C)–(H) show the kinetic rate constants of the 4sXB and the 2sXB models at lattice spacings between 30 and 38 nm. Each rate is a section taken from the corresponding display in Figure 3. (0.24 MB PDF) [file pcbi.1001018.s002.pdf]

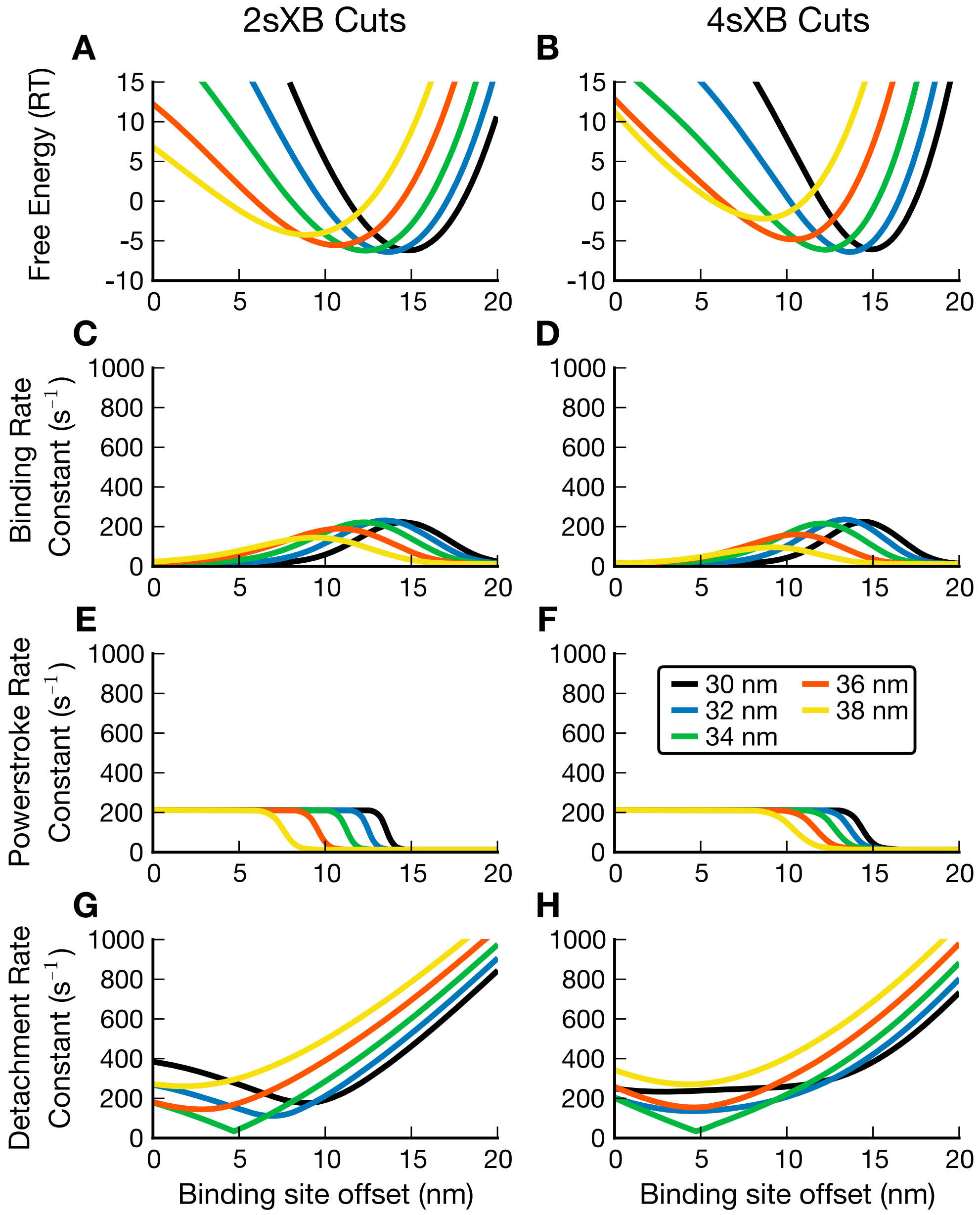

Figure S2: Cross-bridge free energy and kinetics at multiple lattice spacings (A)–(B) show the free energies of the 4sXB and the 2sXB models at lattice spacings between 30 and 38 nm. (C)–(H) show the kinetic rate constants of the 4sXB and the 2sXB models at lattice spacings between 30 and 38 nm. Each rate is a section taken from the corresponding display in Figure 3.
